# Synthesis and Characterization of the Properties of (1−x)Si_3_N_4_-xAl_2_O_3_ Ceramics with Variation of the Components

**DOI:** 10.3390/ma16051961

**Published:** 2023-02-27

**Authors:** Daryn B. Borgekov, Artem L. Kozlovskiy, Maxim V. Zdorovets, Rafael I. Shakirzyanov, Inesh E. Kenzhina, Dmitriy I. Shlimas

**Affiliations:** 1 Engineering Profile Laboratory, L.N. Gumilyov Eurasian National University, Nur-Sultan 010008, Kazakhstan; 2 Department of General Physics, Satbayev University, Almaty 050032, Kazakhstan; 3 Department of Intelligent Information Technologies, Ural Federal University, 620075 Yekaterinburg, Russia; 4 Advanced Electronics Development Laboratory, Kazakh-British Technical University, 59 Tole bi St., Almaty 050000, Kazakhstan

**Keywords:** ceramics, dispersed nuclear fuel, phase composition, hardening, inert matrices

## Abstract

The aim of this paper is to study the effect of variation in the component ratio of (1−x)Si_3_N_4_-xAl_2_O_3_ ceramics on the phase composition, strength and thermal properties of ceramics. To obtain ceramics and their further study, the solid-phase synthesis method combined with thermal annealing of samples at a temperature of 1500 °C typical for the initialization of phase transformation processes was used. The relevance and novelty of this study lies in obtaining new data on the processes of phase transformations with a variation in the composition of ceramics, as well as determining the effect of the phase composition on the resistance of ceramics to external influences. According to X-ray phase analysis data, it was found that an increase in the Si_3_N_4_ concentration in the composition of ceramics leads to a partial displacement of the tetragonal phase of SiO_2_ and Al_2_(SiO_4_)O and an increase in the contribution of Si_3_N_4_. Evaluation of the optical properties of the synthesized ceramics depending on the ratio of the components showed that the formation of the Si_3_N_4_ phase leads to an increase in the band gap and the absorbing ability of the ceramics due to the formation of additional absorption bands from 3.7–3.8 eV. Analysis of the strength dependences showed that an increase in the contribution of the Si_3_N_4_ phase with subsequent displacement of the oxide phases leads to a strengthening of the ceramic by more than 15–20%. At the same time, it was found that a change in the phase ratio leads to the hardening of ceramics, as well as an increase in crack resistance.

## 1. Introduction

One of the most promising solutions in the field of energy, in particular, nuclear energy, is the creation of new types of nuclear reactors capable of operating at high temperatures, as well as having an increased resource of nuclear fuel burnup [1,2,3]. The most effective way to increase the service life is to use new types of nuclear fuel based on technologies for reprocessing weapons-grade plutonium or increasing the fissile material burnup degree. To achieve these goals, the concept of using dispersed nuclear fuel was previously proposed [4,5]. This fuel consists of fissile nuclear material based on plutonium or uranium dioxide, placed in an inert matrix, which serves not only to absorb and remove heat, but also to protect the fissile material from radiation swelling [6,7,8].

As a rule, to create inert matrices of dispersed nuclear fuel, it is proposed to use oxide or nitride ceramics based on refractory compounds, as well as various composites based on them [9,10,11]. Among all compounds, silicon nitride (Si_3_N_4_) and aluminum oxide (Al_2_O_3_) can be especially distinguished, which have high resistance to mechanical stress, crack resistance and corrosion resistance to aggressive media, good thermal conductivity and insulating properties [12,13,14,15]. At the same time, as shown by a number of experimental studies, ceramics based on aluminum oxide [16] and silicon nitride [17,18] have good resistance to radiation damage and their accumulation, as well as maintaining the stability of properties during long-term irradiation with heavy ions, comparable to fission fragments of nuclear fuel. This is an important factor for candidate materials for inert matrices of dispersed nuclear fuel. It should also be noted that in recent years more and more attention has been paid to research aimed at finding ways to obtain two- or three-component ceramics that combine the properties of each component, which contributes to the creation of unique materials with improved properties [19,20,21,22]. For example, it was shown in [23] that Al_2_O_3_-ZrO_2_ ceramics have an increased resistance to helium swelling under high-dose irradiation, and also that the formation of two phases leads to a decrease in the amount of swelling under high-temperature irradiation. The paper [24] shows the results of a study of thermoluminescence of Al_2_O_3_-BeO ceramics subjected to high-dose irradiation. In [25], the authors studied in detail the properties of MgO-Al_2_O_3_ ceramics under irradiation with fast neutrons. In [26], the results of studies of Y_2_O_3_-Al_2_O_3_-SiO_2_ composites are presented, according to which the proposed compositions have an increased resistance to external influences. Interest in this area of research does not decrease despite the sufficient number of various works, as well as the results obtained, which indicates a high level of prospects for these materials not only in the energy sector, but also in mechanical engineering, engine building, etc.

The choice of a composite based on Al_2_O_3_ and Si_3_N_4_ compounds for consideration as candidate materials for creating inert matrices of dispersed nuclear fuel is due to the following factors. Firstly, both Al_2_O_3_ and Si_3_N_4_ compounds have very high melting points of 2072 °C for Al_2_O_3_ and 1900 °C for Si_3_N_4_, which makes them very promising materials for use in operating conditions at elevated temperatures [27,28,29,30]. Secondly, these compounds are characterized by high wear resistance and hardness, which is actively used in the creation of refractory materials with high heat resistance, resistance to cracking and aggressive media, acids, melts, etc. [31,32,33,34,35]. Thirdly, both Al_2_O_3_ and Si_3_N_4_ compounds have a good reputation for use as structural materials for nuclear power engineering due to their high radiation resistance as well as resistance to external influences. However, in a number of works [36,37], it was shown that under certain types of radiation exposure in ceramics based on silicon nitride, extended defects can form in the form of latent tracks, at a high concentration of which partial amorphization of the damaged near-surface layer can occur [38]. A number of authors attribute the appearance of such effects to the energy losses of interacting particles, as well as the possibility of initializing the polymorphic transformation processes in silicon nitride, due to its phase composition and the presence of several structural modifications [38,39,40]. In turn, ceramics and aluminum oxide powders are in some cases used as protectors to increase resistance to external influences, including radiation damage [41,42,43]. Also, ceramics based on aluminum oxide are good insulators and also have antifriction properties [44]. In this regard, the use of a combination of these Al_2_O_3_ and Si_3_N_4_ compounds opens up opportunities for creating ceramics that have the positive properties of both compound types, which can later be considered as promising candidates for creating dispersed nuclear fuel.

The relevance and novelty of this study consists in a detailed study of the effect of component variation in (1−x)Si_3_N_4_-xAl_2_O_3_ ceramics on their structural, strength, and dielectric properties, as well as the choice of the most optimal compositions for further research related to determining the resistance to radiation damage during irradiation with heavy ions. The choice of (1−x)Si_3_N_4_-xAl_2_O_3_ ceramics with different variations of components as objects of study is due to their prospects for use as a basis for materials of inert matrices of dispersed nuclear fuel, which consist in increased resistance to radiation damage, as well as to destruction processes under external mechanical influences.

## 2. Experimental Part

The synthesis of (1−x)Si_3_N_4_-xAl_2_O_3_ ceramics was carried out using the method of solid-phase grinding of initial powders in specified molar ratios with variation of the components, followed by thermal annealing. Al_2_O_3_ and Si_3_N_4_ powders with a grain size of 1–3 µm and a chemical purity of 99.95% were used as initial components. These powders were purchased from Sigma Aldrich (Sigma Aldrich, Burlington, MA, USA). Solid state grinding was carried out using a PULVERISETTE 6 classic line planetary mill (Fritsch, Berlin, Germany). Grinding conditions: grinding speed—400 rpm, grinding time—30 min, tungsten carbide balls with a diameter of 8 mm were used as grinding media. After grinding, the obtained samples were subjected to thermal annealing in a SNOL muffle furnace (SNOL-term, Tver, Russia) at a temperature of 1500 °C for 5 h, the heating rate was 20 °C/min, and the samples were cooled for 24 h after annealing. Thermal annealing of the samples was carried out on uncompressed powders placed in alundum crucibles. Before and after thermal annealing, the powders were weighed using an analytical balance to determine the change in mass of the samples as a result of thermal sintering. According to the data obtained, after thermal annealing, a decrease in the mass of powders in crucibles by 5–10% was observed, depending on the ratio of the components. Such a change indicates the initialization of phase transformation processes as a result of thermal annealing. The annealing itself was carried out in an air atmosphere. The choice of annealing temperature (1500 °C) is due to the possibility of changing the phase composition of ceramics not only due to the variation of the components used for synthesis, but also the phase transformation processes, which are initiated when a barrier of 0.3–0.5 T_melting_ is reached.

The analysis of the phase composition of composite ceramics was carried out using the method of X-ray phase analysis implemented on a D8 Advance ECO powder diffractometer (Bruker, Berlin, Germany). Registration of diffraction patterns was carried out in the Bragg-Brentano geometry in the angular range of 2θ = 20°–90°, with a step of 0.05° and a spectrum acquisition time of 1 s at a point. X-ray diffraction was taken on annealed loose powders, which were not subjected to additional polishing or grinding to avoid introducing mechanical deformations into the annealed ceramic samples. To identify the phase composition, the PDF-2 database (2016) was used, the phases were refined by the method of comparative analysis of the positions of experimentally obtained diffraction reflections and the positions of lines from the database. Determination of the phase composition and their ratio was carried out using the Rietveld method. To analyze the obtained X-ray diffraction patterns using the Rietveld method, the program code TOPAS v.4.0 (Bruker, Berlin, Germany) was used. Verification of phase determination was more than 95%. The refinement of the crystal lattice parameters was carried out taking into account deformation distortions and substitution effects.

The morphological features and elemental composition of ceramics depending on composition variation were studied using scanning electron microscopy and energy dispersive analysis, implemented on a Hitachi TM3030 microscope (Hitachi, Tokyo, Japan). SEM images and mapping data were taken on loose powders preliminarily dispersed on target holders to obtain a uniform powder layer on the holder surface.

The study of the optical properties of the synthesized ceramics depending on the component variation was carried out using the UV-Vis spectroscopy method implemented using a Specord-250 UV spectrophotometer (Jena Analytic, Berlin, Germany). Recording of UV-Vis spectra was carried out on free-flowing powders using an integrated sphere for collecting spectra. In order to use a small volume of synthesized powders for measurements, we used BaSO_4_, which is an optically transparent powder over the entire measured range. Before measurements, a small volume (less than 0.01 g) of the powder under study was mixed with BaSO_4_ and placed in a special chamber in an integrated measurement sphere. In this case, the reference measurement was performed with placed BaSO_4_ without a sample to avoid the influence of the environment on the measurements. The spectra were recorded in the range from 300 to 1000 nm with a step of 1 nm. The determination of the band gap (E_g_) for the corresponding edge of fundamental absorption was performed by constructing Tauc’ plots with subsequent interpretation using Expression (1):

(1) $$\alpha =A{\left(h\nu -E_{g}\right)}^{1/2}$$

where *A* is a constant and *hν* is the photon energy. The calculation of the linear refractive index (*n^optical^*) was carried out using Formula (2):

(2) $$\frac{\left[{\left(n^{optical}\right)}^{2}-1\right]}{\left[{\left(n^{optical}\right)}^{2}+2\right]}=1-\sqrt{\frac{E_{g}}{20}}$$

The calculation of optical transmission (*T^optical^*) and refraction loss (*R^loss^*) was performed using Formulas (3) and (4):

(3) $$T^{optical}=\frac{2\left(n^{optical}\right)}{{\left(n^{optical}\right)}^{2}+1}$$

(4) $$R^{loss}={\left(\frac{\left(n^{optical}\right)-1}{\left(n^{optical}\right)+1}\right)}^{2}$$

To calculate the value of molar refraction (*R^molar^*), Expression (5) was applied:

(5) $$R^{molar}=\left(\frac{{\left(n^{optical}\right)}^{2}-1}{{\left(n^{optical}\right)}^{2}+2}\right)V^{molar}$$

The metallization criterion was determined using Equation (6):

(6) $$M=1-\frac{R^{molar}}{V^{molar}}$$

The metallization criterion characterizes the degree of ordering of ceramic or glassy ceramics, which, when increased, indicates that the optical properties of the material are characteristic of ordered structures, and in the case of a decrease in the criterion value, the properties are characteristic of amorphous glassy structures.

The determination of the static permittivity value was carried out using Formula (7):

(7) $$\varepsilon^{static}={\left(n^{optical}\right)}^{2}$$

The dielectric properties of the resulting ceramics were studied by impedance spectroscopy. The synthesized powders were mixed with a binder, which was obtained from an aqueous solution of polyvinyl alcohol. The residual concentration of polyvinyl alcohol was about 5%. The resulting mixtures (press powders) were pressed into tablets with a diameter of 11 mm and a thickness of 1 mm. Silver paste was applied to both sides, then the tablets were dried for ~2 h at 60 °C. The frequency spectra were measured on a HIOKI IM3533-01 RLC meter (Hioki, Tokyo, Japan) in the frequency range of 100–200,000 Hz.

The hardness of the samples, which reflects the strength properties of ceramics with varying composition, was studied using a LECO LM700 microhardness tester (LECO, Tokyo, Japan). To determine the resistance to single compression, an LFM-L test machine (Walter + bai AG, Lohningen, Switzerland) was used. Measurement of hardness, as well as resistance to single compression, was carried out on pressed samples in the form of tablets, 1 mm thick and 11 mm in diameter. The samples were pressed using a steel mold, the maximum pressure at which the samples were pressed into tablets was 300–400 MPa. The samples were pressed using a cylindrical steel mold. The pressed samples were held in a mold under pressure for 1 h; the pressing process itself was carried out at room temperature in an air atmosphere without heating. After pressing, the specimens were polished to eliminate surface roughness that could affect indentation. The hardness measurement was carried out with a load on the indenter of 500 N, the number of points for measurements was chosen from 25 to 30, in order to collect statistics. A Vickers pyramid was used as an indenter.

## 3. Results and Discussion

Interest in ceramic materials based on oxide and nitride compounds applicable as inert matrices of dispersed nuclear fuel is due to their high strength and thermal parameters. However, despite the large number of different studies, the search for new types of composites for this area is still relevant in view of the large number of different variations in ceramic compositions. One of the important criteria for evaluating the applicability of various composite ceramics is a detailed study of their phase composition, as well as its changes with varying ceramic components. The most accurate answer to the question concerning the variation of the phase composition with a change in the composition of the initial mixtures subjected to thermal annealing at high temperatures can be the use of the X-ray phase analysis method.

**Figure 1:**
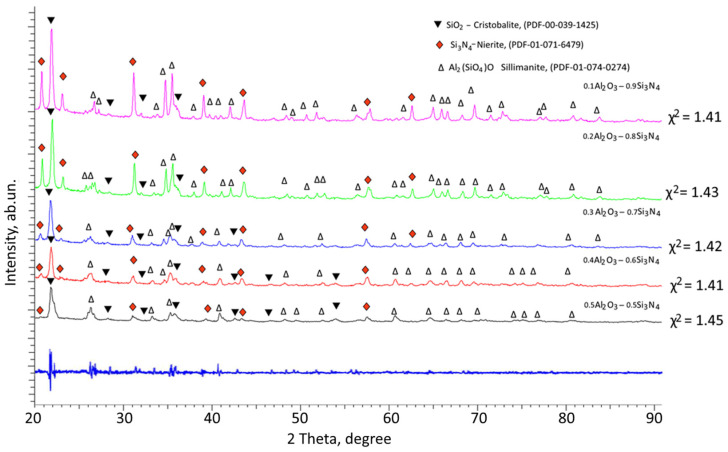
Results of X-ray phase analysis presented as X-ray diffraction patterns of the studied (1−x)Si_3_N_4_-xAl_2_O_3_ ceramics depending on the variation of the components.

Figure 1 shows the data of X-ray phase analysis of the studied (1−x)Si_3_N_4_-xAl_2_O_3_ composite ceramics depending on the component variation. The general view of the presented X-ray diffraction patterns indicates the polycrystalline nature of the ceramics. At the same time, the analysis of the change in the shape of the diffraction lines, as well as the positions of the maxima of the reflections, depending on the ratio of the components, showed the absence of the appearance of any new reflections characteristic of new phases. This indicates that the main processes of phase transformations with variation in the ratio of the components consist in changing the ratio of the established phases. According to the phase analysis of the obtained diffraction patterns, in the structure of ceramics, regardless of the ratio of the components, the presence of three phases is observed: the tetragonal SiO_2_ phase close to the Cristobalite structure, the hexagonal Si_3_N_4_ phase similar to the Nierite structure, and the orthorhombic Al_2_(SiO_4_)O or (Al_2_O_3_)(SiO_2_) phase with the Sillimanite structural motif. At the same time, no reflections characteristic of the Al_2_O_3_ phases were found, which indicates that during thermal annealing, the Al_2_O_3_ phase is transformed into the Al_2_(SiO_4_)O phase by partial replacement of Al ions by Si ions. The appearance of the SiO_2_ phase is characteristic of the decomposition of the main Si_3_N_4_ phase with subsequent transformation into the SiO_2_ phase when Si ions combine with oxygen. At the same time, the analysis of the weight contributions of various phases showed that in all cases of variation in the ratio of components, the dominant phase is SiO_2_ in the composition of ceramics.

**Figure 2:**
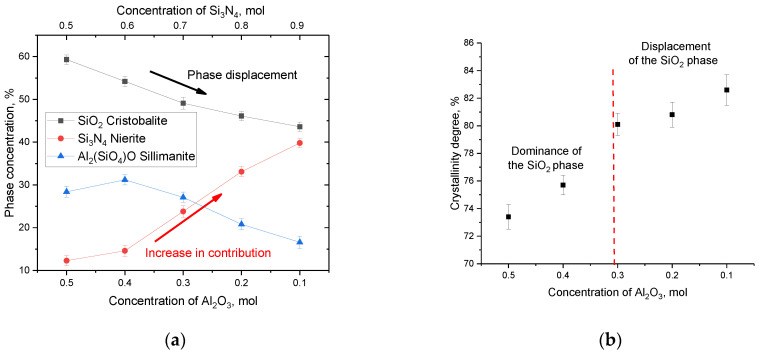
(**a**) Diagram of the phase composition of (1−x)Si_3_N_4_-xAl_2_O_3_ ceramics depending on the variation of the components; (**b**) Graph of the change in the crystallinity degree of xAl_2_O_3_-(1−x)Si_3_N_4_ ceramics depending on the variation of the components (the values of the degree of crystallinity were refined using the Rietveld method).

Figure 2a shows the results of estimating the weight contributions in (1−x)Si_3_N_4_-xAl_2_O_3_ ceramics depending on the variation of the components obtained from the analysis of X-ray diffraction patterns. According to the obtained data, a decrease in the content of Al_2_O_3_ in the composition of ceramics first leads to an insignificant increase in the contribution of the Al_2_(SiO_4_)O phase, which can be explained by the fact that during phase formation a decrease in the SiO_2_ phase is observed with an increase in the contribution of the Si_3_N_4_ phase. In turn, the increase in the contribution of the Si_3_N_4_ phase can be explained by the fact that in the phase formation process, with an increase in the silicon nitride concentration in the annealed mixture, incomplete decomposition of Si_3_N_4_ occurs, followed by its transformation into SiO_2_. These processes of the Si_3_N_4_→SiO_2_ type are possible in the case of thermal annealing in an oxygen-containing environment or in an air atmosphere at temperatures above 1000–1300 °C. With an increase in the Si_3_N_4_ concentration to 0.7 mol and higher, there is a sharp increase in the contribution of the Nierite phase and a gradual displacement of the Cristobalite phase, the content of which decreases to less than 50%. Also, the dominance of the Si_3_N_4_ phase is accompanied by the displacement of the Al_2_(SiO_4_)O phase, the content of which, in the case of an Al_2_O_3_ concentration of 0.2–0.1 mol, is less than 20%.

Figure 2b presents data on the change in the crystallinity degree, reflecting the ratio of contributions from the ordered structure and structurally disordered or amorphous-like regions. The crystallinity degree was calculated by comparative analysis of the areas of diffraction reflections and background radiation characteristic of X-ray diffraction in amorphous or highly disordered inclusions.

An analysis of the data obtained showed that in the case when the SiO_2_ phase dominates in the ceramic structure, the crystallinity degree is no more than 75–76%, which indicates the presence of a sufficiently large number of structurally disordered regions that are formed during the synthesis as a result of phase formation. At the same time, an increase in the Si_3_N_4_ phase contribution leads to an increase in the crystallinity degree above 80%, which indicates that the ceramic structure becomes more perfect and ordered. Such an increase in the crystallinity degree indicates an increase in structural ordering in the synthesized samples due to recrystallization processes, as well as a decrease in the contributions of deformation distortions and stresses arising in the process of mechanochemical synthesis. Also, a change in the crystallinity degree can be associated with a change in the phase ratio, which is characteristic of the displacement of the Cristobalite phase at concentrations of the Si_3_N_4_ component above 0.7 mol. According to the data on the change in the phase ratio, it was found that the displacement of the SiO_2_ phase leads to an increase in the density of ceramics from 2.69 g/cm^3^ at an Al_2_O_3_ concentration of 0.5 mol to 2.81–2.82 g/cm^3^ at an Al_2_O_3_ concentration of 0.2–0.1 mol. At the same time, an increase in the Si_3_N_4_ phase from 12% to 14% at a concentration of 0.4 mol Al_2_O_3_ leads to an increase in the density of 1.7% with is 2.74 g/cm^3^.

**Figure 3:**
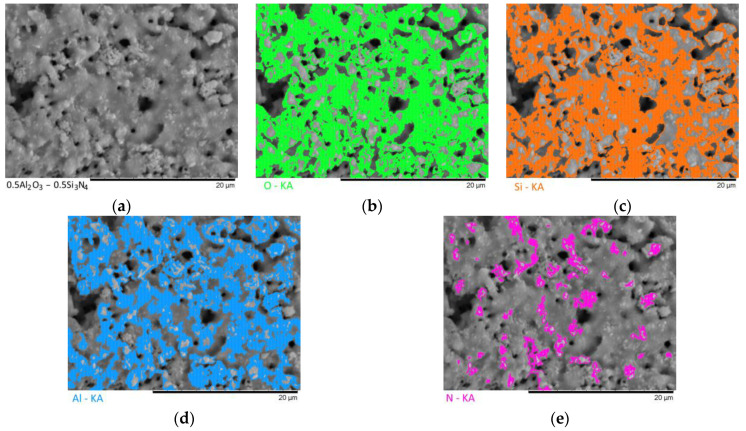
Results of morphological studies and mapping of a ceramic sample with an Al_2_O_3_ content of 0.5 mol: (**a**) SEM image; (**b**) oxygen distribution results; (**c**) silicon distribution results; (**d**) aluminum distribution results; (**e**) nitrogen distribution results.

**Figure 4:**
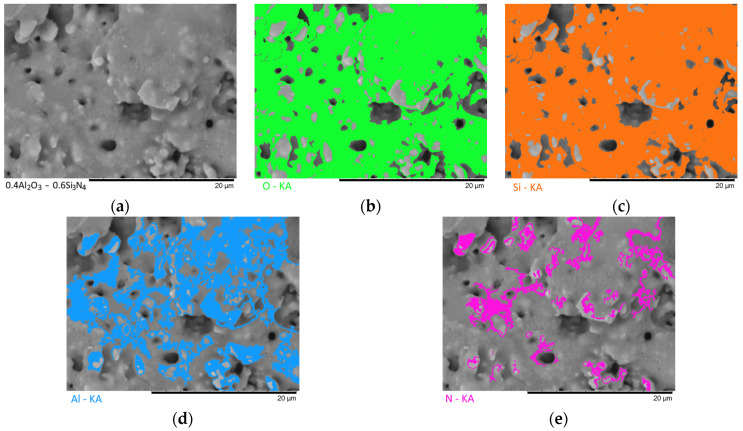
Results of morphological studies and mapping of a ceramic sample with an Al_2_O_3_ content of 0.4 mol: (**a**) SEM image; (**b**) oxygen distribution results; (**c**) silicon distribution results; (**d**) aluminum distribution results; (**e**) nitrogen distribution results.

**Figure 5:**
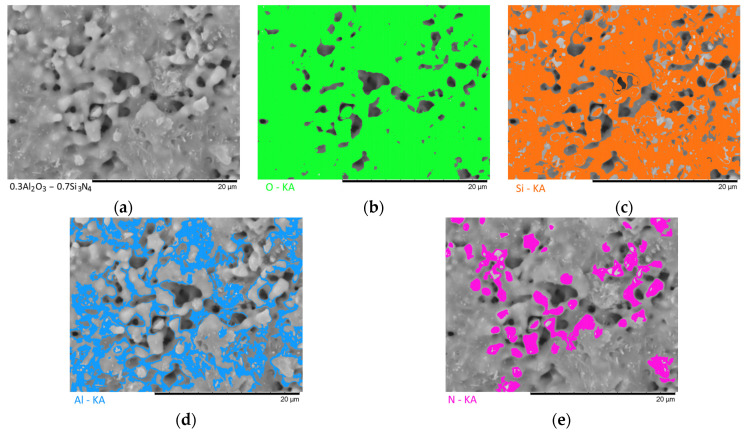
Results of morphological studies and mapping of a ceramic sample with an Al_2_O_3_ content of 0.3 mol: (**a**) SEM image; (**b**) oxygen distribution results; (**c**) silicon distribution results; (**d**) aluminum distribution results; (**e**) nitrogen distribution results.

**Figure 6:**
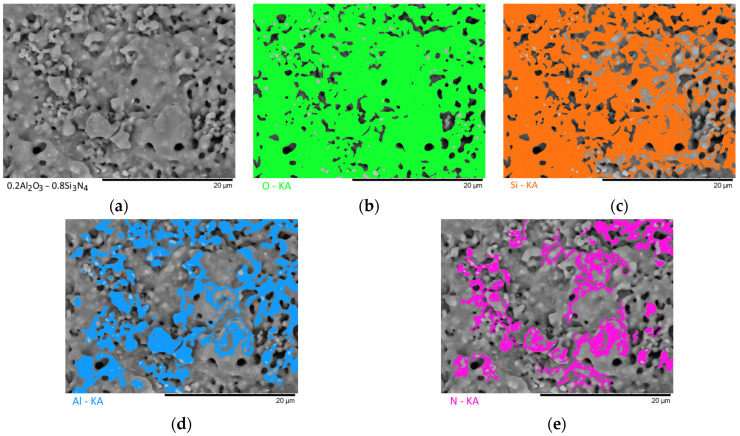
Results of morphological studies and mapping of a ceramic sample with an Al_2_O_3_ content of 0.2 mol: (**a**) SEM image; (**b**) oxygen distribution results; (**c**) silicon distribution results; (**d**) aluminum distribution results; (**e**) nitrogen distribution results.

**Figure 7:**
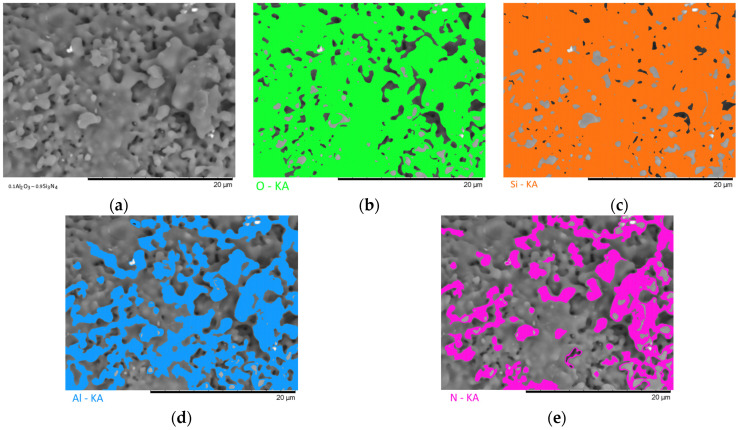
Results of morphological studies and mapping of a ceramic sample with an Al_2_O_3_ content of 0.1 mol: (**a**) SEM image; (**b**) oxygen distribution results; (**c**) silicon distribution results; (**d**) aluminum distribution results; (**e**) nitrogen distribution results.

Figure 3, Figure 4, Figure 5, Figure 6 and Figure 7 show the results of the morphological features of the studied ceramics depending on the variation of the components, as well as the data on the uniformity of the distribution of elements in the composition of samples, performed using the energy dispersive analysis method. According to the data of energy dispersive analysis, the main elements in the samples under study are oxygen, silicon, aluminum, and nitrogen is contained to a lesser extent, and the distribution of nitrogen and aluminum has a pronounced grain structure. The data in Figure 3, Figure 4, Figure 5, Figure 6 and Figure 7 are presented in order to reflect changes in the distribution of elements in the structure of the obtained samples and to indicate that when the ratio of the components changes, the formation of particles of two types containing different elements is observed. In turn, this may indicate that different phases can be present in the structure of the synthesized ceramics.

In the case of a variation of the Al_2_O_3_ component equal to 0.5–0.4 mol, the presence of a large number of grains (approximately 20–25% of the total amount) containing aluminum, silicon, and oxygen in their composition is observed in the ceramic structure, which is typical for the Al_2_(SiO_4_)O phase. Also, in the composition of ceramics, a small content of grains containing only nitrogen and silicon is observed, which is typical for the Si_3_N_4_ phase. Thus, analyzing the morphological features of the obtained structures, we can conclude that the obtained ceramics are the main matrix of SiO_2_ with inclusions in the form of Al_2_(SiO_4_)O and Si_3_N_4_ grains. At the same time, the structure and shape of grains containing a high content of aluminum, silicon and oxygen corresponds to the shape of grains characteristic of Cristobalite.

An increase in the content of Si_3_N_4_ in ceramics leads to an increase in the concentration of grains characteristic of this phase, as well as a slight change in their shape and size, due to the formation of dendrite-like inclusions in the structure of ceramics.

Summarizing the obtained data of morphological studies of synthesized ceramics, we can draw the following conclusions. Firstly, a change in the ratio of the Al_2_O_3_ and Si_3_N_4_ components leads to minor changes in the shape of the grains, as well as their sizes. Secondly, the data obtained from the mapping results indicate that the studied ceramics are a mixture of grains of different phases, including formations in the form of agglomerates or dendrites containing grains of different phases interconnected. At the same time, the dendritic form of inclusions becomes more pronounced with a decrease in the content of the Al_2_O_3_ component.

**Figure 8:**
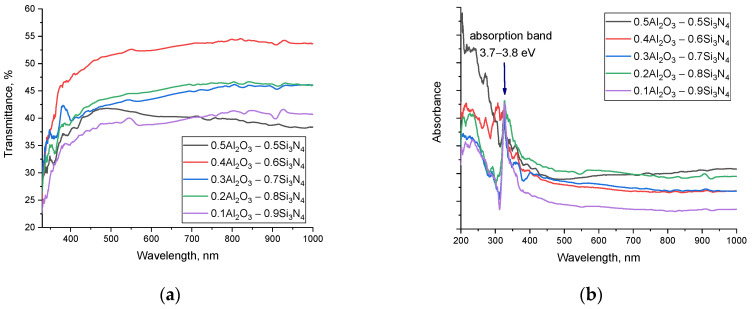
(**a**) Results of UV-Vis transmission spectra of (1−x)Si_3_N_4_-xAl_2_O_3_ ceramics versus component variation; (**b**) Results of UV-Vis absorption spectra of (1−x)Si_3_N_4_-xAl_2_O_3_ ceramics depending on component variation.

Figure 8 shows the results of the evaluation of the optical properties of the synthesized (1−x)Si_3_N_4_-xAl_2_O_3_ ceramics obtained using UV-Vis spectroscopy methods. The data are presented as dependences of changes in the transmission spectra and absorption spectra of the samples under study, reflecting the effect of the phase ratio on the optical properties of ceramics. The general view of the presented transmission spectra (see Figure 8a) is characterized by a fundamental absorption edge in the region of 320–350 nm, as well as an almost constant transmission value within 35–50% in the visible (400–700 nm) and near-IR range (700–1000 nm). In this case, a change in the concentration of the components leads to a shift in the fundamental absorption edge, a change in which indicates a change in the electron density, band gap, and linear refractive index.

An analysis of the absorption spectra showed that a change in the phase ratio in the composition of ceramics leads to the appearance of an additional absorption band in the region of 3.7–3.8 eV, the intensity of which increases with an increase in the contribution of the Si_3_N_4_ phase in the composition of ceramics. The formation of additional absorption bands is due to the formation of absorbing centers in the structure, the appearance of which is associated with a change in the electron density due to a change in the phase composition. The change in the fundamental absorption edge, which also characterizes the change in the band gap, also indicates a change in the electron density. The shift of the fundamental absorption edge to the long-wavelength side indicates a change in interband transitions, which is also reflected in the formation of additional absorption bands.

Based on the obtained UV-Vis spectra using the Tauc plotting technique, the values of the band gap, as well as the linear refractive indices, were determined. The results are presented in Table 1, also using the calculation Formulas (1)–(7), the main optical characteristics were calculated, the values of which reflect the change in the optical properties of ceramics.

As can be seen from the analysis of the data obtained, the formation of the stable Si_3_N_4_ phase in the structure and the subsequent increase in its contribution leads to an increase in the band gap and a decrease in the refractive index, which indicates a change in the optical density and electronic structure of the ceramics. Reducing the refractive index leads to an increase in throughput in the entire visible and IR range, as well as a decrease in reflection losses.

**Figure 9:**
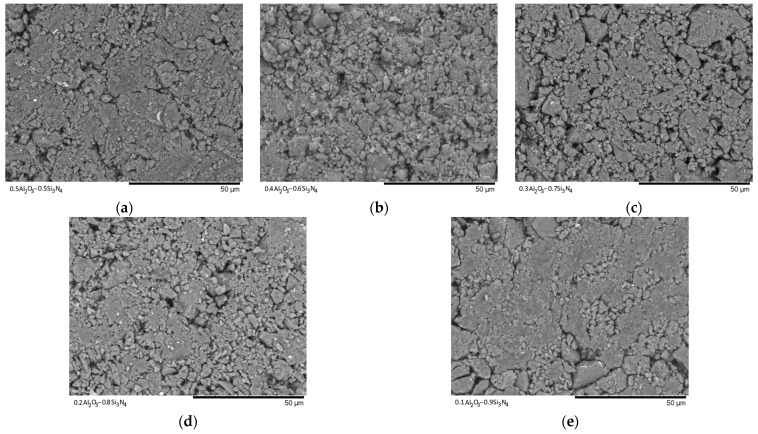
SEM images of synthesized ceramics in Secondary electron image mode: (**a**) 0.5Al_2_O_3_–0.5Si_3_N_4_; (**b**) 0.4Al_2_O_3_–0.6Si_3_N_4_; (**c**) 0.3Al_2_O_3_–0.7Si_3_N_4_; (**d**) 0.2Al_2_O_3_–0.8Si_3_N_4_; (**e**) 0.1Al_2_O_3_–0.9Si_3_N_4_.

**Figure 10:**
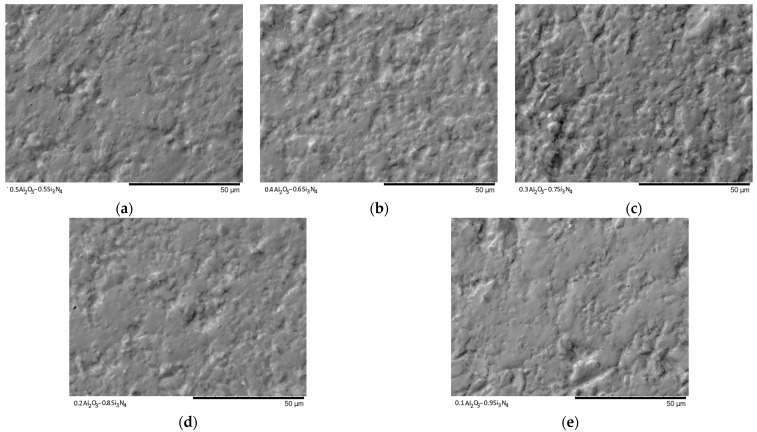
SEM images of synthesized ceramics in the Backscattered electron image mode: (**a**) 0.5Al_2_O_3_–0.5Si_3_N_4_; (**b**) 0.4Al_2_O_3_–0.6Si_3_N_4_; (**c**) 0.3Al_2_O_3_–0.7Si_3_N_4_; (**d**) 0.2Al_2_O_3_–0.8Si_3_N_4_; (**e**) 0.1Al_2_O_3_–0.9Si_3_N_4_.

Figure 9 and Figure 10 show the SEM images of the synthesized ceramics after pressing them into tablets for examination in the Secondary electron image (SE) and Backscattered electron image (BSE) modes, which reflect the change in the morphology of the ceramic topography.

As can be seen from the presented data on the morphological features of the samples after pressing to measure the mechanical strength and resistance to cracking, with a change in the concentration of the components, a significant change in the relief of the grains, as well as their sizes, is observed, with the formation of large agglomerates.

**Figure 11:**
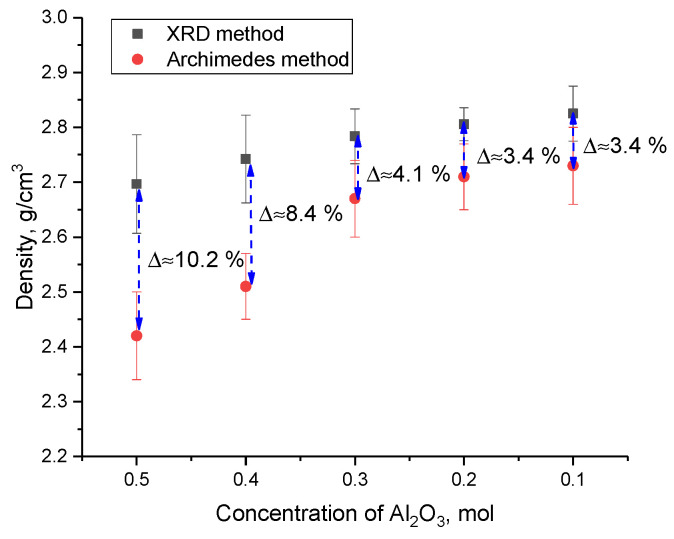
These changes in the density of ceramics, depending on the variation of the components.

Figure 11 shows the data on changes in the density of ceramics calculated using the X-ray phase analysis method, as well as the standard method of Archimedes, which was applied to ceramics pressed into tablets.

According to the obtained data on the change in density values obtained using two methods, a significant difference is seen in the values at high concentrations of Al_2_O_3_ (more than 8–10%). This difference in absolute density (XRD data) and relative density (data obtained using the Archimedes method) is due to the presence of pores and a large number of grain boundaries associated with fine grains. In this case, the change in the phase composition, as well as, as can be seen from these morphological features, leads to a decrease in the difference in the values of the density values obtained using various methods.

**Figure 12:**
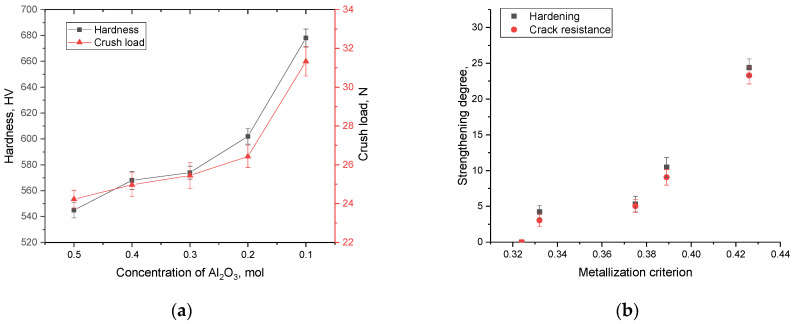
(**a**) Dependence of the change in the strength characteristics of (1−x)Si_3_N_4_-xAl_2_O_3_ ceramics with variation of the components; (**b**) Dependence of the change in hardening characteristics on the criterion of metallization of ceramics.

Figure 12 shows the results of changes in strength characteristics, reflecting the resistance of ceramics to external influences and cracking during indentation and single compression, depending on the variation of the components in the composition. The general trend in the change in the strength properties of ceramics shows that a change in the ratio of components, followed by displacement of the SiO_2_ and Al_2_(SiO_4_)O phases, leads to an increase in the hardness of ceramics and crack resistance. At the same time, the highest indicators of the strength properties of ceramics are typical for samples in which the content of the Al_2_(SiO_4_)O phase is less than 20%, which gives an increase in hardness and strength by more than 20%. At the same time, these changes are most pronounced when determining the resistance to cracking of ceramics under a single compression.

Figure 12b shows the change in hardening characteristics, expressed as a percentage, depending on the criterion for metallization of ceramics, calculated based on the change in optical characteristics. According to the dependences obtained, it can be seen that the greatest hardening is observed in the case of an increase in the metallization criterion by more than 0.37, which leads to an increase in crack resistance by more than 10–25%. In the case of the value of the metallization criterion, which is less than 0.37, the strengthening of ceramics is no more than 3–5%. Such hardening of samples, depending on the metallization criterion, can be explained by the fact that a change in the phase ratio in ceramics leads to the displacement of disordered regions in the composition of ceramics, which is also evidenced by data on the crystallinity degree of the samples. At the same time, these changes are most pronounced in the case when the displacement of the Al_2_(SiO_4_)O phase is observed in the composition of ceramics and its content is less than 20%.

**Figure 13:**
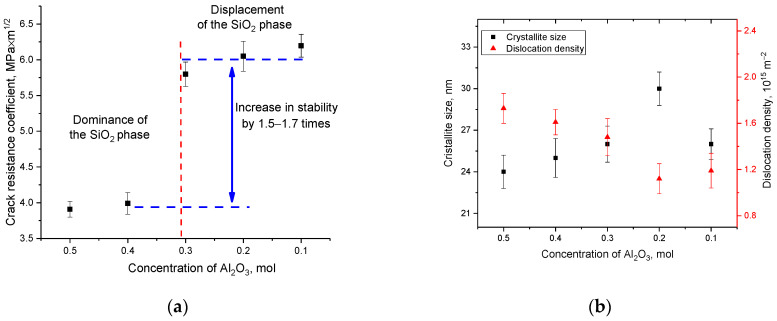
(**a**) Results of evaluation of the change in crack resistance depending on the variation of components in the composition of ceramics; (**b**) Results of the change in crystallite sizes and dislocation density depending on the variation of the components in the composition of ceramics.

Based on changes in hardness values and evaluation of indenter prints, the parameters of ceramic crack resistance were determined depending on the variation in the composition of the components, the results of which are presented in Figure 13a. As can be seen from the presented data, the displacement of the SiO_2_ phase and a decrease in the content of the Al_2_(SiO_4_)O phase leads to an increase in resistance to cracking and an increase in the strength properties of ceramics. At the same time, these changes are non-linear, and the maximum increase is observed for samples in which the content of the SiO_2_ and Si_3_N_4_ phases is close to the equilibrium value, with a low content of the Al_2_(SiO_4_)O phase.

As is known, one of the most common hardening criteria is a change in the dislocation density in samples, an increase in which at small crystallite sizes leads to a significant increase in the resistance of ceramics or alloys to external influences. At the same time, the variation of the phase composition can also affect the change in the strength and stability of ceramics. Figure 13b shows the results of the change in the dislocation density of the samples under study, obtained on the basis of data on changes in the grain size of ceramics. The dislocation density (*δ*) was calculated using the expression: δ=1D2, where *D* is the grain size of the samples under study. The use of this formula is primarily due to the fact that the crystallite size obtained from the analysis of X-ray diffraction patterns is used to calculate the dislocation density. This makes it possible to determine the dislocation density in the entire volume of the structure, and not only in the near surface, as is done when using optical methods for estimating the dislocation density. As can be seen from the data presented, the variation in the composition of ceramics leads to insignificant changes in the crystallite size, which in turn affects the dislocation density. However, these changes in the dislocation density do not have a significant effect on the hardening of ceramics. Thus, we can conclude that in the case of (1−x)Si_3_N_4_-xAl_2_O_3_ ceramics, the main effect in hardening is exerted by a change in the phase ratio, as well as the metallization factor and the crystallinity degree, while a change in the dislocation density does not affect hardening.

**Figure 14:**
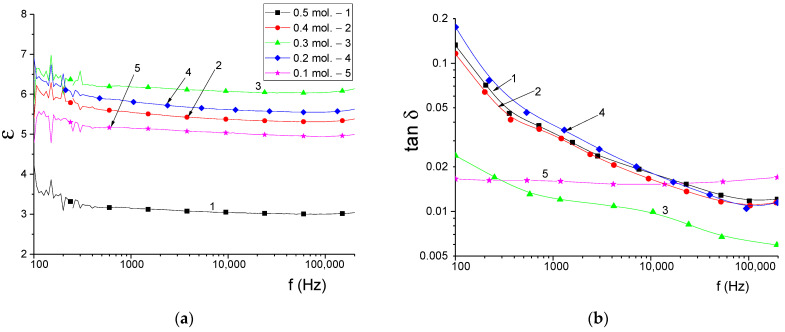
(**a**) Frequency spectra of the permittivity of the obtained samples; (**b**) Frequency spectra of the dielectric loss tangent of the obtained samples.

An important parameter for polycrystalline ceramics is the permittivity ε, the value of which largely determines the interaction of ceramics with radiation. Usually, materials are characterized by dielectric permittivity ε and dielectric loss tangent tan δ, which shows what part of the energy of the electric alternating field is converted into thermal [45]. The dielectric permittivity of ceramics depends on many factors: microstructure (grain size, morphology and porosity), phase composition, defects, substitutions. Since X-ray studies and optical measurements have shown that the obtained polycrystalline powders have a high crystallinity degree, the dielectric properties of the synthesized powders will be mainly determined by the microstructural characteristics and phase composition. During the synthesis of ceramics, as mentioned earlier, phase transformations occur, as a result of which silicon oxide SiO_2_ is the dominant phase. The results of changing the dielectric characteristics are shown in Figure 14. It is reported that SiO_2_ with a tetragonal lattice (Crystobalite) has excellent dielectric parameters with a rather low value of ε ≈ 3.70–4.43 depending on the crystalline state (single crystal, amorphous substance) [46,47,48]. Polycrystalline ceramics based on Si_3_N_4_ are characterized by a higher dielectric constant, the value of which for nonporous samples is in the range of 7.0–8.5 [49,50,51]. The data of dielectric parameters measured for Al_2_(SiO_4_)O with rhombic crystal lattice show that the dielectric constant has a value of 4.71–4.80. For all the above compositions, the value of the dielectric loss tangent is 2 × 10^−3^–15 × 10^−3^, which causes the use of SiO_2_, Si_3_N_4_ ceramics as radio-transparent ceramics. As can be seen from the figure, the curves do not show pronounced frequency dependence, which is typical for Si_3_N_4_ and SiO_2_ ceramics. Figure 14b shows the frequency dependences of the dielectric loss tangent of the samples under study.

In contrast to the frequency dependences of ε, the frequency spectra of tan δ exhibit pronounced frequency dispersion except for the 0.1 mol Al_2_O_3_ sample. Table 2 shows the measurement results of the dielectric parameters of the samples.

It can be seen that the values of ε do not coincide with the calculated static permittivity. Moreover, since the dielectric properties of multiphase ceramics depend on the dielectric properties of individual phases and their concentration, samples with a lower initial concentration of Al_2_O_3_ should have higher values of ε. This is expected because the dielectric constant of silicon nitride, the content of which increases, is almost 2 times higher than that of silicon oxide and Al_2_(SiO_4_)O. This dependence of properties is associated with the features of the microstructure of ceramics (the presence of pores), which can be seen from the results of scanning electron microscopy. For example, the minimum value of the permittivity for a sample of 0.5 is explained by high porosity and a large scatter in grain sizes. Higher values of ε are observed for a more uniform ceramic microstructure for samples of 0.4, 0.3 mol Al_2_O_3_. The increased values of tan δ at low frequencies are associated with a slight influence of the polymer binder. The values of tan δ at the limiting frequency of measurements correspond in order to the measured values in the microwave region in other works cited above, since at high frequencies the molecular chains of the polymer do not contribute to the polarization processes. For samples of 0.1 and 0.2 mol Al_2_O_3_, it can also be noted that the increased value of dielectric losses with the morphology of the samples, in particular, the heterogeneity of the grain size distribution. The sample with 0.3 mol Al_2_O_3_ is characterized by the lowest dielectric loss and the highest value of dielectric permittivity. Thus, the value of the permittivity and dielectric losses in the frequency range 2–100 Hz is largely determined by the microstructure of the samples and, to a lesser extent, by the composition of the ceramics.

The obtained results of the study related to the development of the technology for obtaining (1−x)Si_3_N_4_-xAl_2_O_3_ depending on the variation of the ceramic components have very great prospects in practical terms, not only as materials for inert nuclear fuel matrices, but also as materials for stereolithography or high-strength compounds. Thus, the compositions of ceramic powders obtained can be used as a basis for expanding 3D printing and manufacturing ceramic parts [52]. In turn, the results of the strength properties of the synthesized ceramics are in good agreement with the data of [53], which shows the prospect of using such ceramics as high-strength cutting tools with high wear resistance. At the same time, the proposed method for obtaining ceramics, with the possibility of a controlled phase composition, makes it possible to expand the range of practical application of these ceramics, and the obtained dependences of the effect of the phase composition on the strength and dielectric properties will expand the understanding of the properties of these composite ceramics.

## 4. Conclusions

The article is devoted to the study of structural, morphological, optical, strength and dielectric properties of (1−x)Si_3_N_4_-xAl_2_O_3_ ceramics obtained by solid-phase synthesis with varying the ratio of components. According to the data of X-ray phase and energy-dispersive analysis, it was found that the studied samples are a matrix consisting of SiO_2_ (Crystobalite) with inclusions in the form of Al_2_(SiO_4_)O and Si_3_N_4_ grains. During the studies, it was found that a decrease in the content of Al_2_O_3_ in the composition of ceramics leads to the displacement of Al_2_(SiO_4_)O grains from the structure, with the formation of samples close to the equilibrium composition, which are a matrix of SiO_2_ with inclusions of Si_3_N_4_ grains. During evaluation of the strength properties of the studied ceramics, it was found that the increase in crack resistance and hardness is due to a change in the phase composition, as well as an increase in the contribution of the Si_3_N_4_ phase. According to the results of impedance spectroscopy in the frequency range 100–100,000 Hz, the values of the dielectric constant of the obtained ceramics were measured. The value of ε was in the range of 3.03–6.07, while the dielectric loss tangent was in the range of 0.006–0.038. The value of the permittivity and dielectric losses in the above frequency range is largely determined by the microstructure of the samples and, to a lesser extent, by the composition of the ceramics.

Results obtained make it possible to predict the area of practical application of the synthesized ceramics under conditions of increased mechanical loads, as well as external influences, including ionizing radiation.

## Figures and Tables

**Table 1:** Data on changes in optical characteristics.

Parameter	Concentration of Al_2_O_3_, mol				
	0.5	0.4	0.3	0.2	0.1
*Band gap, eV*	2.109	2.211	2.814	3.034	3.637
*Linear refractive index*	2.691	2.650	2.449	2.387	2.243
*R_loss_*	0.209	0.204	0.184	0.168	0.147
*T_optical transmission_*	0.653	0.661	0.689	0.712	0.743
*Static dielectric constants*	7.241	7.022	6.245	5.698	5.031
*R_molar_*	69.79	69.15	66.74	65.81	62.41
*Metallization criterion*	0.324	0.332	0.375	0.389	0.426

**Table 2:** Results of measurements of dielectric parameters ε, tan δ of the obtained samples.

Concentration of Al_2_O_3_, mol	ε	tan δ		
		1 kHz	10 kHz	100 kHz
0.5	3.03	0.034	0.018	0.012
0.4	5.38	0.032	0.016	0.011
0.3	6.07	0.012	0.010	0.006
0.2	5.62	0.038	0.018	0.011
0.1	5.05	0.016	0.015	0.016

## Data Availability

Not applicable.
